# A whole-body diffusion MRI normal atlas: development, evaluation and initial use

**DOI:** 10.1186/s40644-023-00603-5

**Published:** 2023-09-14

**Authors:** Therese Sjöholm, Sambit Tarai, Filip Malmberg, Robin Strand, Alexander Korenyushkin, Gunilla Enblad, Håkan Ahlström, Joel Kullberg

**Affiliations:** 1 Department of Surgical Sciences, Uppsala University, Uppsala, Sweden; 2 Department of Information Technology, Uppsala University, Uppsala, Sweden; 3 Antaros Medical AB, Mölndal, Sweden; 4 Department of Immunology, Genetics and Pathology, Uppsala University, Uppsala, Sweden

**Keywords:** Whole-body DWI, ADC, Normal atlas, Voxel-wise analysis, Precision, Lymphoma, Automated segmentation

## Abstract

**Background:**

Statistical atlases can provide population-based descriptions of healthy volunteers and/or patients and can be used for region- and voxel-based analysis. This work aims to develop whole-body diffusion atlases of healthy volunteers scanned at 1.5T and 3T. Further aims include evaluating the atlases by establishing whole-body Apparent Diffusion Coefficient (ADC) values of healthy tissues and including healthy tissue deviations in an automated tumour segmentation task.

**Methods:**

Multi-station whole-body Diffusion Weighted Imaging (DWI) and water-fat Magnetic Resonance Imaging (MRI) of healthy volunteers (n = 45) were acquired at 1.5T (n = 38) and/or 3T (n = 29), with test-retest imaging for five subjects per scanner. Using deformable image registration, whole-body MRI data was registered and composed into normal atlases. Healthy tissue ADC_mean_ was manually measured for ten tissues, with test-retest percentage Repeatability Coefficient (%RC), and effect of age, sex and scanner assessed. Voxel-wise whole-body analyses using the normal atlases were studied with ADC correlation analyses and an automated tumour segmentation task. For the latter, lymphoma patient MRI scans (n = 40) with and without information about healthy tissue deviations were entered into a 3D U-Net architecture.

**Results:**

Sex- and Body Mass Index (BMI)-stratified whole-body high b-value DWI and ADC normal atlases were created at 1.5T and 3T. %RC of healthy tissue ADC_mean_ varied depending on tissue assessed (4–48% at 1.5T, 6–70% at 3T). Scanner differences in ADC_mean_ were visualised in Bland-Altman analyses of dually scanned subjects. Sex differences were measurable for liver, muscle and bone at 1.5T, and muscle at 3T. Volume of Interest (VOI)-based multiple linear regression, and voxel-based correlations in normal atlas space, showed that age and ADC were negatively associated for liver and bone at 1.5T, and positively associated with brain tissue at 1.5T and 3T. Adding voxel-wise information about healthy tissue deviations in an automated tumour segmentation task gave numerical improvements in the segmentation metrics Dice score, sensitivity and precision.

**Conclusions:**

Whole-body DWI and ADC normal atlases were created at 1.5T and 3T, and applied in whole-body voxel-wise analyses.

**Supplementary Information:**

The online version contains supplementary material available at 10.1186/s40644-023-00603-5.

## Background

Diffusion-Weighted Imaging (DWI) can be used to study the movement of water molecules, governed mainly by tissue cellularity, cell membrane integrity and fluid viscosity. It provides both visual and, by calculation of the Apparent Diffusion Coefficient (ADC), quantitative evaluations of tissue microcellular architecture. One usage area is in the diagnoses and longitudinal monitoring of cancer, with the ADC being a promising cancer imaging biomarker shown to inversely correlate with tumour cellularity [1]. With the introduction of the Diffusion-Weighted whole-body Imaging with Background body signal Suppression (DWIBS) technique, whole body tumour analysis has been made possible [2, 3]. High tumour to background contrast is obtained by using high diffusion sensitising gradient imaging, Short-TI Inversion Recovery (STIR) for fat suppression and a free breathing scan allowing for multiple signal averaging [4].

Statistical atlases have been constructed for a number of anatomical sites, providing population-based descriptions from multiple healthy controls and/or patients into single 3D representations using image registration. This approach has most extensively been used in the brain, for which single- or multi-modal representations of the healthy and diseased brain have been thoroughly investigated [5]. Averaging imaging features across individuals have identified group-specific patterns of brain structure. This approach has also been assessed in other single organ sites such as the heart [6], lung [7] and prostate [8]. For whole-body imaging, the approach of population-based atlases has been less studied. Medical image registration algorithms are in general purposely designed for specific body parts, with whole-body image registration being more challenging due to large inter-subject anatomical variations. Whole-body image registration algorithms have however been described [9–11] and a normal atlas for multi-modal ^18^F-Fluorodeoxyglucose (FDG) Positron Emission Tomography (PET)/Magnetic Resonance Imaging (MRI) suggested [12]. The advantage of a whole-body atlas framework includes the potential of studying systemic diseases such as metabolic syndrome and cancer [13], without reducing the analysis to a set of pre-defined Regions of Interest (ROIs). There is also the potential for voxel-wise comparisons between patient scans and normative data.

Studies of healthy tissue ADC have been highlighted as vital for establishing the precision of ADC measurements [14]. Healthy tissue ADC assessments from whole-body DWI have been reported [15, 16], but are scarce.

Whole-body DWI allows cancer monitoring across the whole body, and is commonly evaluated qualitatively from high b-value images and quantitatively from lesion-wise ADC measurements. Using this approach, early response assessment has been shown possible in e.g. lymphoma [17]. Moving beyond the standard lesion-wise assessment of ADC, it has been suggested that other more advanced metrics can be used for improved tumour evaluation and response assessment. These include the total Diffusion Volume (tDV) for assessment of global disease burden [18] and histogram analysis of a single lesion or the total tumour burden for assessment of tumour spatial heterogeneity [19, 20]. For advanced measurements to be feasible, automated workflows for tumour segmentation are needed. Due to the high contrast between tumour and background, high b-value imaging is promising for this task. Blackledge et al. developed a semi-automated lesion segmentation approach using computed DWI (cDWI) [18]. To reduce image noise, this method was extended by Gatidis et al., who presented the voxel-wise cDWI (vcDWI) [21]. Information about voxel ADC is utilised in the vcDWI calculation, giving an improvement in signal and contrast to noise ratio, and a reduction in T2 shine-through effects. For fully automated tumour segmentation, supervised Convolutional Neural Network (CNN) based methods have recently been developed, with the most widely used architecture being the U-Net [22]. It has successfully been used to segment tumours in many cancers, including whole-body FDG PET applications. For DWI, U-Net has been used in single organ applications such as automatic segmentation of ischemic brain injury [23] and brain tumours [24]. To the best of our knowledge, it has yet to be applied to whole-body DWI. Large initiatives for automated tumour quantification in whole-body DWI using machine learning have however been described in e.g. myeloma [25].

This work aims to create, evaluate and employ a normal atlas of whole-body DWI and ADC of healthy volunteers scanned at 1.5T and 3T. The atlas is created using deformable image registration and evaluated by establishing whole-body ADC values of healthy tissues, including test-retest ADC measurements, comparison of ADC across field strengths and assessments of the effect of age and sex on ADC. We further employ the normal atlas in an automated tumour segmentation task, together with a deep learning approach, to investigate whether information about healthy tissue deviations could be advantageous in this task.

## Methods

### Subjects

In this prospective study, 45 healthy adult volunteers were recruited between January 2019 and February 2020 (mean age 45.3 ± 14.0 years, range 25–77 years, 23 females and 22 males). Ethics approval was obtained from the Uppsala regional ethics committee (Dnr 2017/524) and signed informed consent was obtained from all subjects before participation. Basal data including age, sex, height and weight were collected at time of imaging. The medical history of each subject was recorded, as well as any current medications. Exclusion criteria were contraindications to MR imaging (i.e. pacemaker, implanted devices, claustrophobia), contraindications to Buscopan administration, pregnancy and breast-feeding. Subjects with metal implants and known disease affecting the normal appearance of imaging (e.g. tumour disease) were not included. All subjects were asked to participate in scanning on two scanners. Five subjects per scanner were imaged using a test-retest protocol, with imaging repeated after a short toilet break.

To evaluate the atlas in an automated tumour segmentation task, a dataset of relapsed/refractory large B-cell lymphoma patients were included. This dataset contains 24 patients scanned longitudinally before and after therapy using Chimeric Antigen Receptor (CAR) T-cells. Patients scanned on PET/MRI and with measurable FDG-avid disease were included in the current study (n = 16, median age 63 years, range 37–71 years, 9 females and 7 males). A total of 40 PET/MRI scans were available, with each patient being scanned at 1–5 time points. Ethics approval was obtained from the Uppsala regional ethics committee (Dnr 2017/449) for retrospective data analysis.

### Imaging

Imaging was performed with a 1.5T scanner (Achieva, Philips Healthcare, Best, The Netherlands, gradient system: 33 mT/m maximum amplitude, 180 T/m/s maximum slew rate) and a 3T scanner (Signa PET/MR, GE Healthcare, Milwaukee, WI, USA, gradient system: 44 mT/m maximum amplitude, 200 T/m/s maximum slew rate). Station-wise scan parameters are shown in Table 1. Healthy volunteers were scanned at 1.5T and 3T, while lymphoma patients were scanned at 3T. Bowel preparation consisted of ≥ 4 h fasting and, to minimise peristaltic movements, an intramuscular injection of 20 mg Hyoscine Butylbromide (Buscopan). Head volume and phased array body coils were used for signal reception, with subjects in a head first supine position. Multi-station whole-body images were acquired axially in free breathing using a water and fat MRI sequence and a diffusion-weighted spin echo Echo Planar Imaging (EPI) sequence with STIR fat suppression. Scan coverage was head to mid-thighs, corresponding to five or six stations per subject. For healthy volunteers, an EPI sequence with reverse phase encoding was also acquired to enable DWI geometric distortion correction using the Reverse Polarity Gradient (RPG) method [26, 27]. For lymphoma subjects, FDG PET imaging was included in the examination.

**Table 1:** Station-wise MR image acquisition parameters at 1.5T and 3T

	1.5T			3T		
Sequence	DWI		T1w Dixon	DWI		T1w Dixon
Sequence details	EPI, AP	EPI, PA	mDIXON	EPI, AP	EPI, PA	LAVA-Flex
Respiration	Free breathing		mixed*	Free breathing		mixed*
Slices per station (n)	40		96	38		100
Overlapping slices (n)	3		8	5		23
Fat suppression	STIR		-	STIR		-
Parallel imaging factor	2.5		2	2		2
TR (ms)	5600		5.5	3500		4.1
TE (ms)	73		1.7/3.7	61.7		1.7
TI (ms)	180		-	245.9		-
Flip angle	90		15	90		12
FOV (mm)	440 × 361		400 × 400	440 × 352		500 × 450
Acquired matrix	128 × 103		200 × 200	128 × 96		256 × 212
Slice thickness (mm)	6		5	6		5
Bandwidth (Hz/pxl)	2719		587	1953		1302
b-values (s/mm^2^)	0, 50, 400, 900	0, 50	-	0, 50, 400, 900	0, 50, 900**	-
NSA	2, 2, 4, 9	2, 2	1	2, 2, 4, 9	2, 2, 4	1
Acquisition time	4:29 min	0:50 min	19 s	3:09 min	1:35 min	16 s

The reverse phase encoding EPI sequence (EPI, PA) was acquired for healthy volunteers and not for lymphoma patients. *Breath-hold for neck, chest and abdomen stations, free breathing for head, pelvis and leg stations. ** b = 900 s/mm^2^ added to get an equal number of segments for the AP and PA acquisitions, enabling the same TR to be set. SS-EPI = single shot EPI, AP = anterior-posterior phase encoding, PA = posterior-anterior phase encoding, NSA = number of signal averages, bandwidth = receiver bandwidth.

ADC maps were calculated station-wise from b = 50, 400 and 900 s/mm^2^ images using a mono-exponential log-linear least square fit [28]. Water Fraction (WF) and Fat Fraction (FF) images were calculated from water and fat MRI [29]. Acquired stations were composed into single whole-body volumes by removing an equal number of overlapping slices from adjacent stations. Intensity blending was not performed. An experienced radiologist (HA) screened all healthy imaging data for incidental findings.

### Image registration

**Fig. 1:**
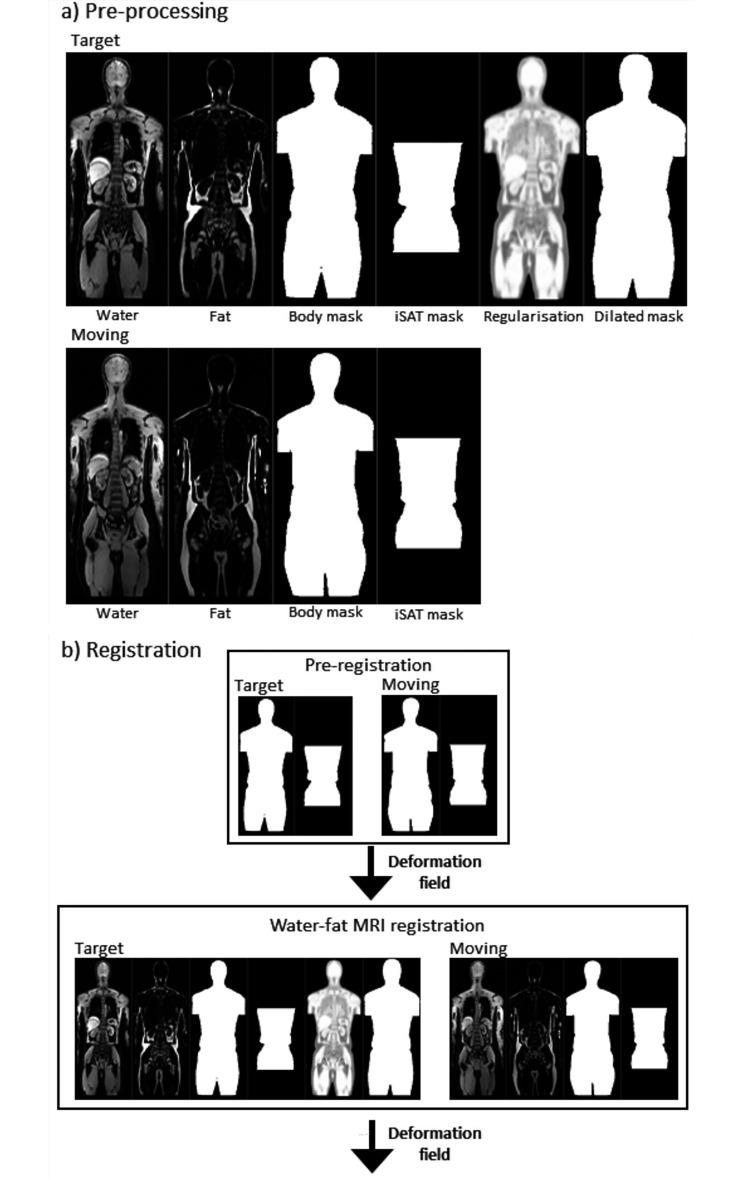
Image pre-processing **(a)** and registration pipeline **(b)**, where iSAT mask corresponds to an inside SAT mask

An image registration pipeline was setup to spatially align whole-body water and fat MR images (Fig. 1) using the open source deform package [30]. In deform, deformable image registration is performed using a graph-cut based method, with a Gaussian smoothing multi-resolution strategy [9]. The registration algorithm utilises a patch-based setup, in which overlapping subsets of the 3D volume is registered and the results then combined. Tissue-specific regularisation weights are used, applied according to voxel-wise FF and WF content. As previously described [10], it is beneficial to set a higher regularisation for lean tissue (high WF) compared to adipose tissue (high FF), allowing for a larger inter-subject difference and higher elasticity of adipose tissue.

Subjects were stratified according to sex and body mass index (BMI). Two male and two female reference subjects were chosen, one corresponding to the healthy weight BMI range (BMI < 25 kg/m^2^) and one corresponding to the overweight BMI range (BMI ≥ 25 kg/m^2^). The remaining healthy subjects’ water and fat MR images were registered to the relevant male or female reference spaces in a pre-processing and image registration pipeline as illustrated in Fig. 1.

In the pre-processing step, binary masks and a regularisation weight map were generated. To separate the body from background, binary body masks were automatically created using thresholding and standard morphological operations. Arm removal was needed due to non-standard arm positioning. Due to the large Subcutaneous Adipose Tissue (SAT) variability in the cohort, binary inside SAT masks were created using an active learning 2D U-Net [22]. After training, user input was needed to mark the start and end slice for the segmentation (armpit and minor trochanter). Furthermore, binary dilated body masks were created in reference space. The dilated body mask defined the region within which the registration cost function was calculated, making the registration faster by background removal. By using a dilated body mask, body edge information was passed to the registration algorithm, giving improved registration results. Compared to previous implementations of the registration pipeline [10, 12], the current pipeline employed a direct image registration method with a regularisation weight map created from reference subject WF and FF images.

The registration was performed in two-steps; pre-registration, and water and fat MRI registration. The pre-registration step included deformable registration of body and inside SAT masks. This step gave a rough alignment of whole-body volumes, with the resulting deformation field used as a starting guess in the following step. For the main registration, input data included water and fat MRI, binary masks, the regularisation weights map and the pre-registration deformation field. Optimal registration parameters were evaluated by calculating the Dice score between fixed and registered moving body masks, number of discontinuities in the Jacobian determinant map and inverse consistency in terms of vector magnitude error, as well as visual image quality assessments.

Taking advantage of the inherent co-registration of simultaneously acquired water and fat MRI and DWI, whole-body DWI and ADC data were transferred to the relevant reference space using the final deformation fields.

### Normal atlases

The registered healthy volunteer data were combined into male and female normal atlases of normal and overweight BMI. For the DWI and ADC images, normal atlas versions with and without geometric distortion correction were created. The distortion corrected images were calculated using an open source framework [26, 31], as previously described [27].

### ADC evaluation

Whole-body ADC was assessed for healthy volunteers. ADC was measured by manual Volume of Interest (VOI) segmentations of ten healthy tissues in 3DSlicer [32]. Tissues were selected as to span the whole body: parietal white matter, cerebellar white matter, liver (segment VI), spleen, kidneys, psoas muscle, vertebral body (L1-L5), pelvic bone (body of ilium), femur and thigh muscle. Multi-slice ROIs were manually placed in the tissue of interest by a medical physicist (TS), with three consecutive axial slices segmented for all tissues in male and female reference spaces, and with access to all imaging data. Circular ROIs were used, except for the spleen, kidneys and pelvic bone for which oval or crescent shaped ROIs were used. Right and left ROIs were used for white matter, kidneys, muscle, pelvic bone and femur, and then grouped. Additional File 1 shows representative slices of a reference subject at 3T with ROIs used to calculate ADCs for the ten different tissues. The segmentations were then transferred to each subject’s native space, visually assessed and, if needed, adjusted to exclude tissue borders, major vessels and incidental findings. For each subject and each tissue segmented, the multi-slice ROIs were combined into VOIs. For each VOI the mean ADC (ADC_mean_), median ADC (ADC_median_) and size were extracted from non-distortion corrected data.

Voxel-wise correlation between ADC and age was performed across the whole body in reference space. For this purpose, distortion corrected ADC data from all subjects were transformed to the BMI ≥ 25 kg/m^2^ male or female reference space, as applicable, using the registration pipeline illustrated in Fig. 1.

### Tumour segmentation

For whole-body tumour segmentation, a state of the art 3D U-Net [33] was setup with two different network architectures. The baseline architecture included two input channels for DWI and WF data. For DWI data, it was assessed whether b = 900 s/mm^2^ or vcDWI data gave superior segmentation results according to the foreground Dice score. The second architecture was setup with three input channels: DWI, WF and t-map data. DWI data was the best performing data from the baseline model, while the t-map data included voxel-wise statistical deviations between normal atlas and lymphoma patient data (further described below).

WF data was prepared in range [0 1] and resampled to DW image size using linear interpolation. For b = 900 s/mm^2^ data, the signal intensity of the head station was normalised to the neck station signal intensity using histogram matching of overlapping slices [34] prior to combining stations into whole-bodies. The signal intensity of each whole-body DWI dataset was normalised using scaling to the upper quartile [35], followed by min-max normalisation to bring the data into range [0 1]. Max was set to the 99.99th percentile to avoid normalising to image noise.

For t-map data, the male or female atlas components were registered to the native space of each lymphoma patient (registration pipeline, Fig. 1). Patient data, in the form of smoothed (Gaussian, σ = 1.7 mm) b = 900 s/mm^2^ and vcDWI images, were then compared with the relevant atlas component using a voxel-wise one-sided t-test. This resulted in whole-body maps of p-values and t-scores (t-maps) based on b = 900 s/mm^2^ and vcDWI data. To decide which dataset to include as a third channel in the U-Net model, a few hard-coded rules were used: (i) threshold on p < 0.001, (ii) morphological opening to remove small detected clusters and (iii) removal of clusters with max signal intensity < 95th within-body percentile. To reduce false positives, it was further assessed whether removal of voxels belonging to adipose tissue (FF > 50%) gave improved results. The t-map of the dataset achieving the best mean foreground Dice score was included as a third channel in the 3D U-Net setup. This t-map was mapped from [0 200] to [0 1]. To further highlight tumour regions, it was also assessed whether multiplying the t-map with the corresponding normalised DWI data improved the segmentation performance. This t-map was mapped from [0 30] to [0 1].

Training was performed using five-fold cross-validation (4–9 scans per validation fold). 3D patches of size [x = 192, y = 192, z = 160] voxels were used for training, extracted using a sliding window with an overlap of 0.25 between successive patches. The Dice loss function was used for training optimisation (excluding the background) [36] with Adam optimiser and a learning rate of 1e^− 4^, a weight decay of 1e^− 5^ and a dropout factor of 0.20. Manual reference tumour segmentations were performed by two radiologists in consensus according to Lugano classification guidelines [37] and with access to water and fat MRI, DWI, ADC and FDG PET data.

The metrics extracted during five-fold cross validation were tDV, foreground Dice score, True Positive lesions (TP), False Positive lesions (FP), False Negative lesions (FN), sensitivity and precision. Lesion-wise sensitivity was defined as the number of correctly detected lesions (TP) divided by the total number of lesions (TP + FN), while lesion-wise precision was defined as the number of correctly detected lesions (TP) divided by the total number of detected lesions (TP + FP). Lesion-wise metrics were extracted from reference standard and predicted segmentations by connected components analysis, followed by thresholding on a cluster size of > 0.5 ml. A predicted lesion was counted as a TP for a Dice score > 0.01%, when compared to the reference standard segmentation.

### Statistical analysis

The normal atlases are presented as the voxel-wise mean and percentage Coefficient of Variation (CV) for all atlas components. For healthy tissue VOI-based ADC measurements, summary statistics are presented in the form of mean, standard deviation (SD), median and interquartile range (IQR). Healthy tissue ADC differences due to sex were assessed by independent samples t-tests, while ADC repeatability was assessed using the percentage repeatability coefficient (%RC) for test-retest exams [38]. The effect of scanner on ADC was assessed using Bland-Altman statistics. Multiple linear regression was used to test if age and sex significantly predicted ADC_mean_ for the VOI-based analysis, and Pearson correlation was used to assess the voxel-wise correlation between age and ADC for registered whole-body images in male and female reference spaces.

For the anomaly detection task, summary statistics are given as mean, SD, median and IQR.

Statistical significance was set at p < 0.05 and no correction for multiple comparisons was performed. Statistical analyses were performed using the open-source R software (v3.6.1.) or Python NumPy library.

## Results

### Subjects

Subject demographics are shown in Table 2. Of 45 recruited subject, 38 subjects were scanned at 1.5T (mean age 44.1 ± 15.0 years, 25–77 years, 17 females, 21 males) and 29 subjects were scanned at 3T (mean age 47.9 ± 15.0 years, 27–77 years, 13 females, 16 males). One recruited subject did not complete imaging due to claustrophobia. Current medications included blood pressure medication (n = 4), levothyroxine (n = 4), loperamide (n = 2), diabetes medication (n = 1), acetylsalicylic acid (n = 1) and mesalamine (n = 1). Test-retest scanning was performed for five subjects per scanner, with the median time between the start of each scan being 57 min (range 56–68 min) at 1.5T and 74 min (range 65–98 min) at 3T. Twenty-three subjects were scanned at both field strengths, with a median time of 13 weeks (range 0–40 weeks) between scans.

### Normal atlases

**Fig. 2:**
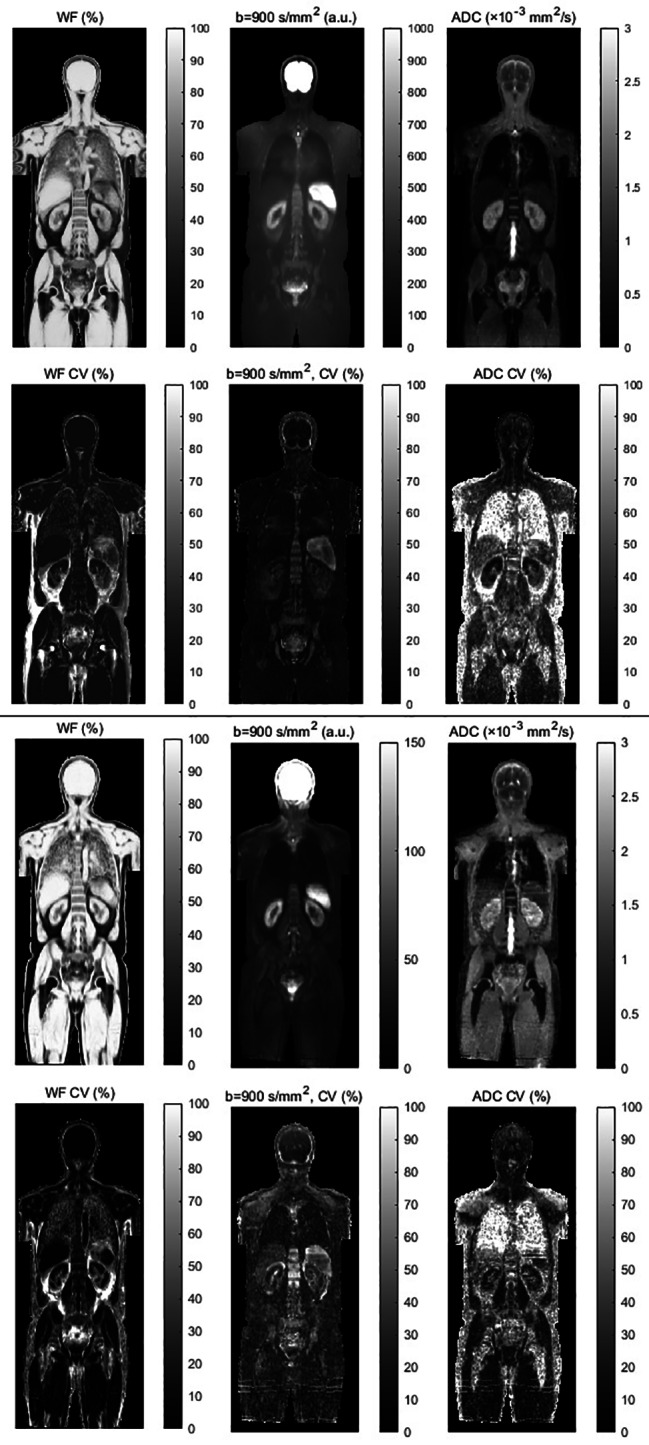
Example atlas images for male BMI ≥ 25 kg/m^2^ healthy volunteers scanned at 1.5T (n = 11, top) and 3T (n = 9, bottom). For each atlas, coronal WF, b = 900 s/mm^2^ and ADC images are shown. The top row corresponds to mean images, while the bottom row corresponds to the percentage CV. Geometric distortion correction was not performed for diffusion images

**Fig. 3:**
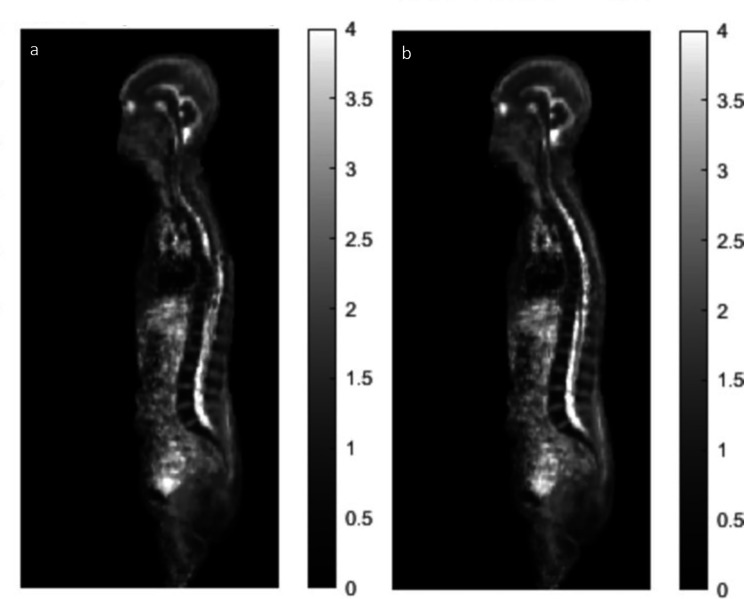
Example of the effect of distortion correction on the 3T atlas. Mean sagittal images for females with BMI < 25 kg/m^2^, showing non-corrected ADC **(a)** and distortion-corrected ADC **(b)** data. The effect of the distortion correction is mainly visible for the spinal column, with discontinuities seen for non-corrected data as marked with an arrow. This is largely corrected for the distortion-corrected data

Healthy subject water-fat MRI data were successfully registered to the relevant reference space. Representative coronal images for the mean and CV of the BMI ≥ 25 kg/m^2^ male atlases at 1.5T and 3T are shown in Fig. 2, while Table 2 shows the number of subjects and characteristics for each atlas component. Movies of all atlas components are provided as Additional Files 2–5. Good registration results were in general obtained, with organ borders well defined. Notably, the signal to noise ratio of b = 900 s/mm^2^ images were reduced at 1.5T compared to 3T, in particular seen for muscle tissue. An example of the effect of incorporating distortion correction in the atlas is shown in Fig. 3.

**Table 2:** Basic characteristics of the healthy subjects included in the atlas

	1.5T					
	Male			Female		
	All	BMI < 25	BMI ≥ 25	All	BMI < 25	BMI ≥ 25
n	21	10	11	17	8	9
Age (years)	44.1 (12.1)	43.7 (13.9)	43.7 (13.9)	44.1 (13.7)	41.9 (12.5)	46.0 (14.5)
Height (cm)	180.3 (5.2)	181.2 (5.3)	179.5 (5.0	167.4 (6.2)	166.1 (4.7)	168.6 (7.0)
Weight (kg)	84.6 (12.0)	75.0 (5.4)	93.3 (9.4)	69.8 (12.7)	59.6 (4.3)	78.9 (10.7)
BMI (kg/m^2^)	26.0 (3.8)	22.9 (1.7)	28.9 (2.8)	24.9 (3.9)	21.6 (1.7)	27.7 (3.0)
	3T					
	Male			Female		
	All	BMI < 25	BMI ≥ 25	All	BMI < 25	BMI ≥ 25
n	16	7	9	13	6	7
Age (years)	43.7 (12.0)	41.4 (13.0)	45.4 (10.8)	53.0 (16.7)	55.3 (15.8)	51.0 (17.1)
Height (cm)	180.9 (3.6)	183.9 (2.5)	178.7 (5.6)	166.9 (4.3)	165.7 (5.0)	168.0 (3.3)
Weight (kg)	84.1 (10.9)	76.9 (5.5)	89.7 (10.7)	69.5 (12.6)	58.2 (5.0)	79.1 (8.3)
BMI (kg/m^2^)	25.7 (3.6)	22.7 (1.7)	28.0 (2.8)	24.9 (4.0)	21.2 (1.1)	28.0 (2.7)

Data is presented as mean (SD).

### ADC evaluation

Summary statistics of ADC_mean_ measured in tissues across the whole body are shown in Table 3, together with the segmented mean VOI size for each tissue, a comparison across sex and test-retest results in the form of %RC. Summary statistics for tissue ADC_median_ are provided as Additional File 6. Overall, small numerical differences were measured between ADC_mean_ and ADC_median_.

**Fig. 4:**
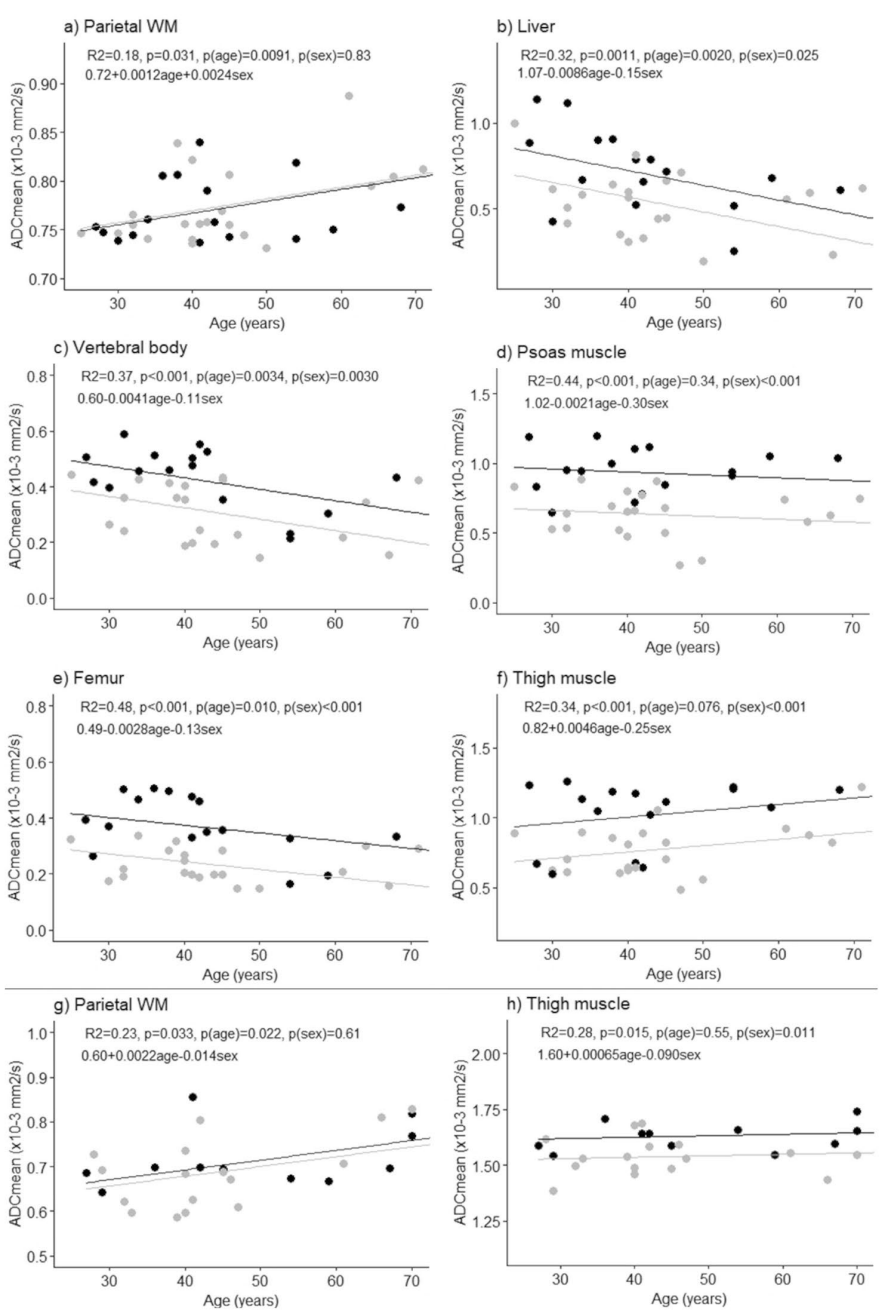
Multiple linear regression fit to tissue ADC_mean_, with age and sex as explanatory variables. Significant results (p < 0.05) are shown for 1.5T **(a-f)** and 3T **(g and h)** data. For each tissue type, R^2^, model p-value, p-value for age, p-value for sex and regression equation are shown (female = 0, male = 1). Male and female measurement points are indicated in grey and black, respectively. Regression lines are shown with sex kept constant, with the grey lines corresponding to males and the black lines to females

Sex differences were mainly measured at 1.5T, with statistically significant differences in ADC_mean_ obtained for the liver (p = 0.045), psoas and thigh muscles (p < 0.001) and bones (femur p < 0.001, vertebral body p = 0.0047). At 3T, a significant sex difference in ADC_mean_ was measured for thigh muscle only (p = 0.0042). The same effect of sex on ADC was seen in the multiple linear regression (Fig. 4). Age and/or sex predicted ADC_mean_ for several healthy tissues, with Fig. 4 showing significant predictions (predictions not reaching statistical significance are shown as Additional File 7). At 1.5T, age was negatively associated with ADC_mean_ for the liver, vertebral body and femur. At both field strengths, a positive association between age and ADC_mean_ was obtained for parietal white matter.

**Fig. 5:**
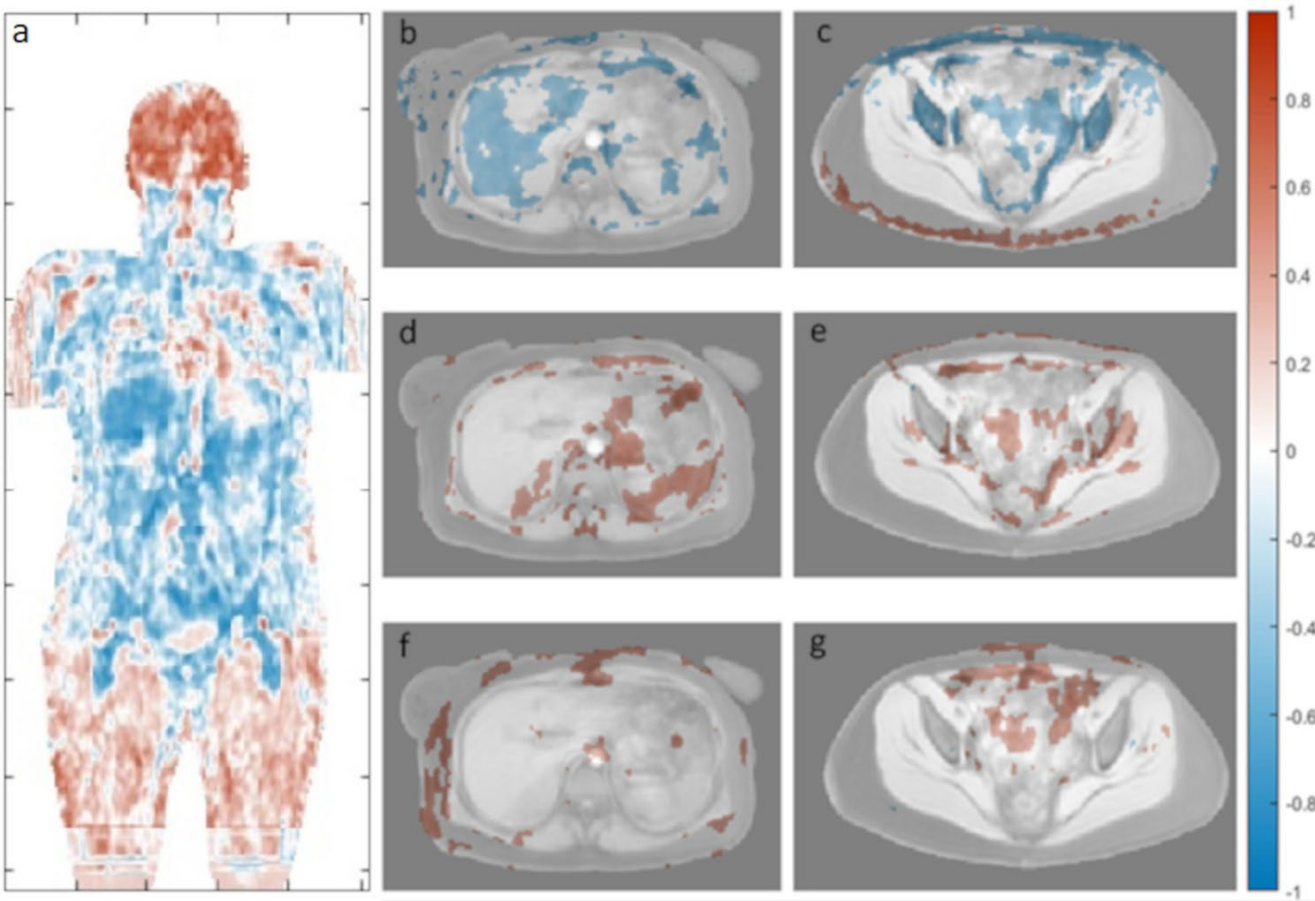
Whole-body R-maps showing voxel-wise correlation between ADC and age for female subjects at 1.5T. The coronal image shows the correlation between ADC and age across the whole body **(a)**. Example axial slices show age correlated with ADC **(b, c)**, FF **(d, e)** and volume **(f, g)** with significant R-values (p < 0.05) overlayed on the atlas mean water image. Distortion corrected ADC data was used to produce the plots

The associations between age and ADC were confirmed in the voxel-wise whole body correlation analysis at 1.5T. As illustrated in Fig. 5 for female subjects, negative correlations between ADC and age were observed for e.g. liver, vertebral bodies and femur, and positive correlations between ADC and age were observed for brain tissue. Voxel-wise correlations between FF and age, and volume and age, are also shown in Fig. 5. Further results of voxel-wise correlation between ADC and age for female and male subjects at 1.5T and 3T are provided as Additional File 8. For males at 1.5T, the voxel-wise correlation between age and ADC showed the same trend as for females, but lower R-values were measured. At 3T, the positive correlation between ADC and brain tissue was visible in the voxel-wise analysis, but body regions had an overall noisy appearance.

Test-retest %RC varied depending on scanner used and tissue assessed (Table 3). Notably, a high repeatability was seen for the brain (%RC < 10% at 1.5T, %RC ≈ 10% at 3T). Muscle tissue had a high repeatability at 3T (psoas muscle 7.3%, thigh muscle 5.9%), but was lower at 1.5T (psoas muscle 48.1%, thigh muscle 34.9%). Also evident from Table 3, was the large differences in ADC_mean_ measured at 1.5T and 3T. Bland-Altman plots including the 23 dually scanned subjects confirmed this (Additional File 9). Statistically significant differences between ADC_mean_ measured at 1.5T and 3T were seen for all assessed tissues except the kidneys, pelvic bone and vertebral bodies.

**Table 3:** ADC_mean_ (×10^− 3^ mm^2^/s) measured in 10 tissues across the whole body at 1.5T (top) and 3T (bottom)

1.5T						
	ADC_mean_ all (n = 38)	VOI size all (n = 38)	ADC_mean_ M (n = 21)	ADC_mean_ F (n = 17)	p-value M vs. F	%RC (n = 5)
Parietal WM	0.77 (0.037)	0.6 (0.2)	0.77 (0.040)	0.77 (0.033)	0.84	7.6
Cerebellar WM	0.67 (0.026)	1.3 (0.4)	0.67 (0.025)	0.67 (0.028)	0.75	4.3
Liver	0.60 (0.24)	8.6 (3.8)	0.53 (0.19)	0.69 (0.26)	**0.045**	48.1
Spleen	0.66 (0.15)	5.4 (2.0)	0.66 (0.14)	0.67 (0.16)	0.87	34.9
Kidney	1.91 (0.19)	7.6 (2.1)	1.88 (0.20)	1.94 (0.17)	0.35	15.0
Vertebral body	0.36 (0.12)	5.9 (1.3)	0.31 (0.10)	0.42 (0.12)	**0.0047**	38.7
Psoas muscle	0.77 (0.23)	4.0 (1.6)	0.63 (0.16)	0.93 (0.18)	**< 0.001**	48.1
Pelvic bone	0.37 (0.10)	3.2 (0.8)	0.37 (0.093)	0.37 (0.11)	0.97	24.9
Femur	0.29 (0.11)	2.5 (0.7)	0.23 (0.060)	0.36 (0.11)	**< 0.001**	45.0
Thigh muscle	0.88 (0.23)	5.5 (1.4)	0.77 (0.17)	1.02 (0.22)	**< 0.001**	34.9
**3T**						
	**ADC**_**mean**_ **all (n = 29)**	**VOI size** **all (n = 29)**	**ADC**_**mean**_ **M (n = 16)**	**ADC**_**mean**_ **F (n = 13)**	**p-value M vs. F**	**%RC (n = 5)**
Parietal WM	0.70 (0.074)	1.6 (0.5)	0.69 (0.077)	0.72 (0.067)	0.23	11.9
Cerebellar WM	0.71 (0.052)	1.4 (0.4)	0.72 (0.054)	0.71 (0.050)	0.63	9.8
Liver	1.24 (0.27)	11.6 (3.3)	1.19 (0.31)	1.30 (0.19)	0.31	17.6
Spleen	0.94 (0.25)	4.6 (1.9)	0.92 (0.25)	0.96 (0.26)	0.72	69.8
Kidney	2.03 (0.20)	7.7 (2.0)	2.04 (0.13)	2.02 (0.26)	0.78	21.2
Vertebral body	0.37 (0.11)	6.5 (1.6)	0.39 (0.11)	0.36 (0.11)	0.44	38.0
Psoas muscle	1.42 (0.067)	5.5 (2.0)	1.40 (0.072)	1.43 (0.057)	0.25	7.3
Pelvic bone	0.37 (0.075)	2.7 (0.7)	0.35 (0.080)	0.39 (0.059)	0.12	36.3
Femur	0.40 (0.076)	2.6 (1.0)	0.37 (0.048)	0.42 (0.095)	0.13	17.8
Thigh muscle	1.58 (0.093)	5.5 (1.5)	1.54 (0.080)	1.63 (0.079)	**0.0042**	5.9

For ADC_mean_ and VOI size (ml), the mean is given with the SD in parentheses. P-values are shown for comparisons between male and female ADC_mean_ values for each tissue type. Statistically significant comparisons are highlighted in bold (p < 0.05). The last column shows test-retest results for each tissue type in the form of %RC. ^M=male, F=female, WM=white matter^.

### Tumour segmentation

**Fig. 6:**
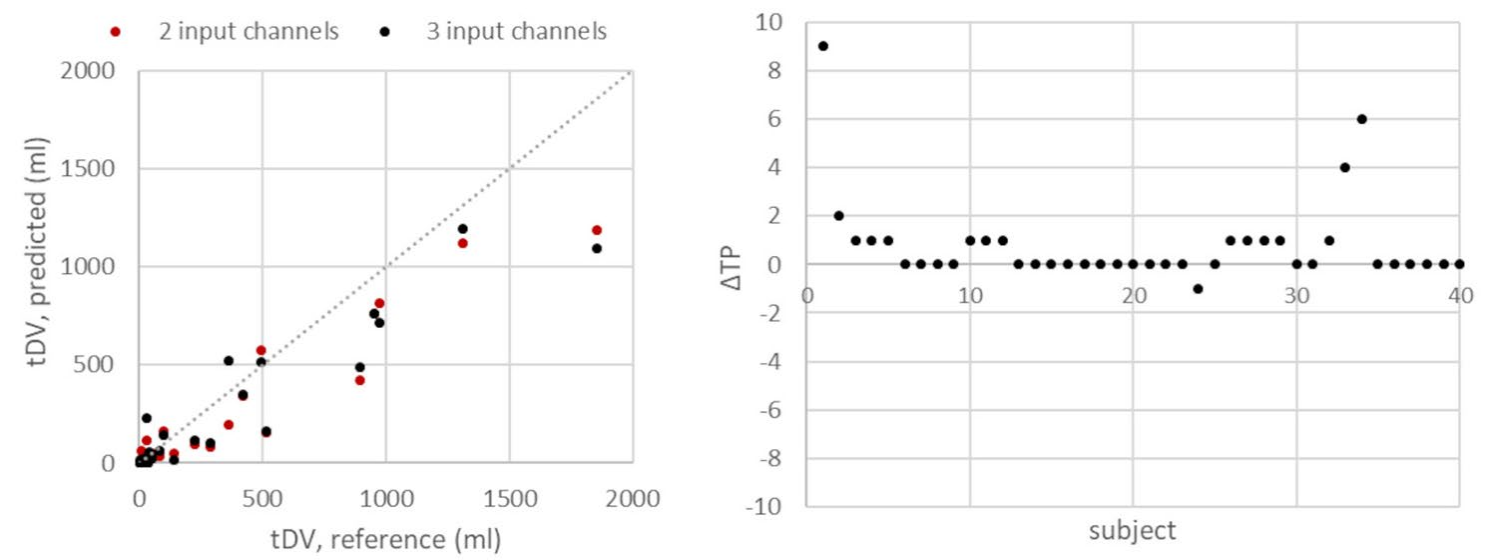
Manual reference and predicted tDV for all patients (left) and the change (Δ) in true positive lesions (TP) with ΔTP = TP(3 input channels)-TP(2 input channels) (right). Plots were produced for the b = 900 s/mm^2^ data for 2 input channels and the t-map×vcDWI data for 3 input channels

Results from the tumour segmentation task are shown in Table 4; Fig. 6, with predicted segmentations shown in Additional File 10. For the baseline model with two input channels, superior segmentation results in terms of Dice score were obtained for b = 900 s/mm^2^ input data (mean/median Dice = 38/40%, sensitivity/precision = 33/65%). For 12 scans (30%), all tumours were detected, with a median Dice score of 74% (range 23–90%) and median reference tDV of 39 ml (range 2-495 ml). For another 12 scans (30%), no tumours were detected, with median reference tDV of 9 ml (range 2–34 ml). The tDV in these patients was in general composed of one or several small tumours. Inferior segmentation results were seen for vcDWI data in terms of Dice score (mean/median Dice = 36/30%). A higher number of TPs were however detected, giving increased sensitivity (36%).

The dataset obtaining the best results in the voxel-wise segmentation approach using the normal atlas was vcDWI data, with FF voxels > 50% removed (mean Dice = 22%). Numerically, the three channel U-Net architecture performed better than the baseline U-Net architecture for all metrics assessed (Table 4, mean/median Dice = 40/45%, sensitivity/precision = 42/69%) as achieved when using the t-map multiplied with vcDWI as input data. For this model, all tumours were detected for 13 scans (33%), with median Dice score of 72% (range 1–92%) and median reference tDV of 33 ml (range 2-495 ml). For 9 scans (23%), no tumours were detected, with median reference tDV of 9 ml (range 2–34 ml). As for the baseline model, the tDV in these patients was in general composed of one or several small tumours.

**Table 4:** Tumour segmentation results for the 3D U-Net with two and three input channels

Architecture	2 input channels		3 input channels	
Input data	WF, b = 900	WF, vcDWI	WF, b = 900, t-map	WF, b = 900, t-map × vcDWI
Dice, mean (SD) [%]	38.3 (32.5)	35.6 (32.5)	39.4 (32.9)	40.4 (32.4)
Dice, median (IQR) [%]	40.0 (74.0)	29.5 (70.5)	43.8 (74.8)	44.6 (69.0)
TP	104/2.6	114/2.9	122/3.1	135/3.4
FN	215/5.4	205/5.1	197/4.9	184/4.6
FP	55/1.4	71/1.8	59/1.5	62/1.6
Sensitivity [%]	32.6	35.7	38.2	42.3
Precision [%]	65.4	61.6	67.4	68.5

The TP, FN and FP are given as total number of tumours/tumours per scan.

In general, predicted tDVs were smaller than manual reference tDVs (Fig. 6, left). The two U-Net architectures detected the same number of TPs for 24 scans (60%), with an increase in the number of TPs seen for 15 scans (38%) when using the U-Net with 3 input channels (Fig. 6, right). For both architectures, datasets with high b = 900 s/mm^2^ signal intensity in bone (n = 6, 15%) gave rise to approximately half of the false positives: n = 26 for 2 input channels with b = 900 s/mm^2^ data, and n = 39 for 3 input channels with t-map×vcDWI data.

## Discussion

In this work, sex- and BMI-stratified whole-body DWI and ADC normal atlases were created at 1.5T and 3T. The atlases were used to study voxel-wise correlations between healthy tissue ADC and age across the whole body, confirming results from a manual segmentation approach. A deep learning based framework for automated tumour segmentation was setup. Statistical deviations between lymphoma subject and normal atlas DW images were shown to numerically improve Dice score, sensitivity and precision of this task.

The normal atlas was created using deformable image registration, building on a previously described method [9] and an open-source software [30]. Instead of performing the image registration in a step-wise manner for bone, adipose and lean tissue, as previously implemented, it has been observed that a voxel-wise regularisation weight map, imposing constraints on the regularisation term, can be included in the registration. This gives an improvement in terms of substantially reducing the number of fold-over artefacts in the Jacobian determinant map and speeds up the registration. In this work, it was further noted that a high variability in BMI poses challenges for the whole-body registration. This problem was minimised by using an inside SAT binary mask. Distortion corrected data was further utilised using the RPG method and was visually shown to improve the geometrical accuracy of the healthy atlases. This has been studied in more detail by others [27, 39].

Measured healthy tissue ADC_mean_ values of this study were in line with those previously reported and obtained from whole-body DWI at 1.5T [15] and 3T [16]. Variations in the selection and number of b-values acquired in different studies however make direct comparisons of ADC difficult. It was possible to perform voxel-wise correlations between ADC and age across the whole body using the atlas. This was in particular evident at 1.5T for female subjects in bone regions and liver. This finding was confirmed by multiple linear regression analysis for manually segmented VOIs and are supported by previous studies for liver [16] and bone marrow [16, 40]. These results were however only seen at 1.5T, potentially due to the smaller number of subjects scanned at 3T. Sex differences in liver, bone and muscle were noted, with female subjects having higher ADC_mean_ compared to men. These findings are also in line with published literature [16]. It has been suggested that ADC changes with age and sex in liver and bone marrow are due to changes in tissue fat content [16], with increased fat content seen for men compared to pre-menopausal women, and with increased age. This could however not be confirmed by the voxel-wise analysis performed in this study, for which a correlation between FF and age was not measurable.

A statistically significant association between ADC and age of parietal white matter was measured at both 1.5T and 3T. Although increased age has been shown to give increased water diffusion in white matter [41], the coarse image resolution in the current study made it difficult to measure white matter only without contamination from cerebrospinal fluid, which most likely affected the results.

Measurement precision is vital for longitudinal studies, to distinguish between measurement noise and true change in a biomarker of interest. ADC repeatability in localised areas of the body such as brain, prostate, breast and liver have been described in the Quantitative Imaging Biomarker Alliance (QIBA) diffusion imaging profile, but measurements of whole-body ADC repeatability is lacking [38]. This study showed that ADC_mean_ repeatability varied in the studied tissues, with the %RC ranging from < 10% for the brain to 48% at 1.5T (liver and psoas muscle) and 70% at 3T (spleen). As such, depending on the position in the body and tissue type, a large percentage change in ADC_mean_ is potentially needed for a true change to be measurable. The measured repeatability is however in the same range as figures reported in the literature. In the QIBA claim statement, %RC in the range 11–47% are given depending on organ. Notably, the %RC of muscle was in this study smaller at 3T compared to 1.5T (6–7% at 3T, 35–48% at 1.5T). This is possibly due to the improved signal to noise ratio offered at 3T. Evident from Fig. 2 and the b = 900 s/mm^2^ atlas image, the level of noise in muscle was larger at 1.5T compared to 3T.

Although the ADC has shown promise as an imaging biomarker, problems linked to its usage include protocol standardisation [14, 42]. Efforts have been made to standardise imaging protocols for obtaining reproducible biomarker measurements in DWI in general [38] and in whole-body scans [43]. Although this was a single-centre study, with acquisition protocols following current guidelines, evaluations of healthy tissues for subjects scanned at both 1.5T and 3T (n = 23) showed that large scanner differences in ADC_mean_ exist for almost all studied tissues. The between-scanner ADC reproducibility, another aspect of precision, was hence low. The time between repeated scans were however long for a subset of subjects (median time 13 weeks, range 0–40 weeks), which is not ideal for measurements of reproducibility. The results however still highlights that large between-scanner differences can exist. A further aspect of standardisation is ADC measurement technique. In this work multiple-slice ROIs were used which has been shown to reduce ADC variability compared to using a single-slice ROI [44]. For reproducibility, the ROI placement used in this work is exemplified in Additional File 1.

An automated tumour segmentation framework was setup for whole-body DWI, in which information about statistical deviations from normality, in the form of t-maps, was shown to numerically improve the prediction performance in terms of foreground Dice score, sensitivity and precision. When creating t-maps, vcDWI data gave the best mean Dice for the predicted segmentations (22%). Usage of t-map data on its own, without a deep learning framework, was not feasible due to a large number of false positive voxels segmented by this approach. The false positives mainly stemmed from DWI signal artefacts, inter-subject signal intensity differences and registration errors. The U-Net was however able to use the t-maps to improve the Dice score and TPs of the predicted segmentations, with only a small increase in FPs.

Overall, automated tumour segmentation for this cohort proved challenging with inferior segmentation results compared to published literature for FDG PET whole body cancer applications [45]. The lymphoma dataset utilised included a limited number of scans and many small tumours with near normal ADC. These tumours were hardly visible in the high b-value diffusion images used for automated tumour segmentation, making this task very challenging. To a large extent, these small tumours were the reason for the U-Net not finding any tumours in a large proportion of scans (n = 12 for 2 input channels, n = 9 for 3 input channels). In an ongoing project, we have achieved a mean Dice of 36% for this cohort when FDG PET and WF data was used as input in the 2 input channels U-Net architecture. This can be compared to a mean Dice of 68% achieved for a larger dataset [46] using the same architecture (unpublished data). A further problem noted with usage of DWI data in general, was the increased signal intensity seen in the bone for a subset of patients, generating a large portion of the false positive tumours.

A general difficulty in MRI is that image signal intensities are arbitrary and do not have tissue-specific meaning. Although quantitative information in the form of the ADC can be obtained, high b-value images are preferentially used for tumour segmentation, and these are of a non-quantitative nature. This can potentially be problematic for the implementation of common post-processing techniques, such as segmentation and quantitation [47]. DWI data suffers from both within- and between-subject signal intensity variations. In general, signal reception is performed with different coils for the head and body, giving large within-subject signal differences. Histogram matching of overlapping slices [34] largely resolved this issue in the current study. Between-subject signal intensity variations for DWI data was to some extent rectified by upper quartile normalisation. Other normalisation methods were tested in this work, including min-max, Z-score and robust Z-score normalisation [35], but did not give improved segmentation results compared to upper quartile normalisation. There are however other signal intensity normalisation techniques that could be assessed, such as histogram-based methods [48].

Future work will include expanding the amount of training data and exploring synergistic effects of using multi-modal FDG PET and DWI data as input in the U-Net architecture. Between-scanner ADC and DWI signal intensity was in this study however shown to be large, and age and sex differences exist for a subset of organs. For multi-centre large-scale deep learning studies, often needed to provide sufficient training data, these factors might prove problematic. Improvements in normalisation techniques, and potentially age- and sex-matched studies, might be needed.

This study includes limitations. The number of subjects included was small, affecting the results of this paper, e.g. ADC repeatability measurements and the U-Net based tumour segmentation task. Due to scheduling issues and healthy subject availability, a long time interval was obtained between scans at 1.5T and 3T for a subset of the subjects, potentially affecting the ADC scanner comparison negatively. The tumour reference segmentations were based on access to FDG PET, DWI and water-fat data. It is possible that the U-Net would have performed better if reference segmentations had been performed on DWI and water-fat data only. Lastly, distortion corrected data for lymphoma patients was not available. This would have been preferable as the geometric distortion of DWI and ADC data can give large discrepancies between diffusion and structural imaging data. In future studies it might be clinically feasible to include distortion correction in a whole-body scanning protocol, as faster sequences for e.g. RPG distortion correction are becoming available [39].

## Conclusion

Sex- and BMI-stratified whole-body DWI and ADC atlases were created at 1.5T and 3T. ADC repeatability varied depending on scanner and tissue assessed and healthy tissue ADC assessments showed large scanner differences, potentially posing challenges for multi-centre data pooling and analysis automation. The atlases were used to study voxel-wise correlations between healthy tissue ADC and age across the whole body, confirming results from a manual segmentation approach. Lastly, a framework for using the normal atlas in an automated tumour segmentation task was presented, with improved segmentation results in terms of Dice score, sensitivity and precision.

### Electronic supplementary material

Below is the link to the electronic supplementary material.

Supplementary Material 1. Additional file 1 contains example ROIs outlined in reference space (AdditionalFile1.pdf).

Supplementary Material 2. Additional files 2-5 contain movie of the male and female normal atlases at 1.5T and 3T (AdditionalFile_2.avi, AddionalFile_3.avi) and 3T (Additionalfile_4.avi, AddionalFile_5.avi).

Supplementary Material 3

Supplementary Material 4

Supplementary Material 5

Supplementary Material 6. Additional file 6 is a table of ADC_median_ measured in 10 tissue across the whole body (AdditionalFile6.pdf)

Supplementary Material 7. Additional file 7 contains a figure showing the multiple linear regression fit to tissue ADC_mean_, with age and sex as explanatory variables, and p > 0.05 (AdditionalFile7.pdf)

Supplementary Material 8. Additional file 8 shows example whole-body R-maps for male and female subjects at 1.5T and 3T (AdditionalFile8.pdf)

Supplementary Material 9. Additional file 9 contains Bland-Altman plots for dually scanned healthy volunteers at 1.5T and 3T (AdditionalFile9.pdf)

Supplementary Material 10. Additional file 10 shows predicted tumours overlayed on b = 900 s/mm^2^ MIPs (AdditionalFile_10.pdf)

## Data Availability

The healthy volunteer datasets collected and analysed, and the code generated, during the current study are available from the corresponding author on reasonable request.

## Abbreviations

%RC: Percentage Repeatability Coefficient
ADC: Apparent Diffusion Coefficient
BMI: Body Mass Index
CAR: Chimeric Antigen Receptor
cDWI: Computed DWI
CNN: Convolutional Neural Network
CV: Coefficient of Variation
DWI: Diffusion Weighted Imaging
EPI: Echo Planar Imaging
FDG: F18-Fluorodeoxyglucose
FF: Fat Fraction
FN: False Negative
FOV: Field of View
FP: False Positive
MIP: Maximum Intensity Projection
MRI: Magnetic Resonance Imaging
PET: Positron Emission Tomography
ROI: Region of Interest
RPG: Reverse Polarity Gradient
SAT: Subcutaneous Adipose Tissue
STIR: Short TI Inversion Recovery
tDV: Total Diffusion Volume
TP: True Positive
vcDWI: Voxel-wise cDWI
VOI: Volume of Interest
WF: Water Fraction
