# Supplementary material for: A whole-body diffusion MRI normal atlas: development, evaluation and initial use
Source: Cancer Imaging. 2023 Sep 14;23:87. doi: 10.1186/s40644-023-00603-5 (PMC10503210; doi:10.1186/s40644-023-00603-5)
Supplement: Supplementary file 1 — Supplementary Material 1. Additional file 1 contains example ROIs outlined in reference space (AdditionalFile1.pdf). [file 40644_2023_603_MOESM1_ESM.pdf]

Additional file 1. Representative slices of a reference subject at 3T showing examples of ROIs used to calculate ADC values.

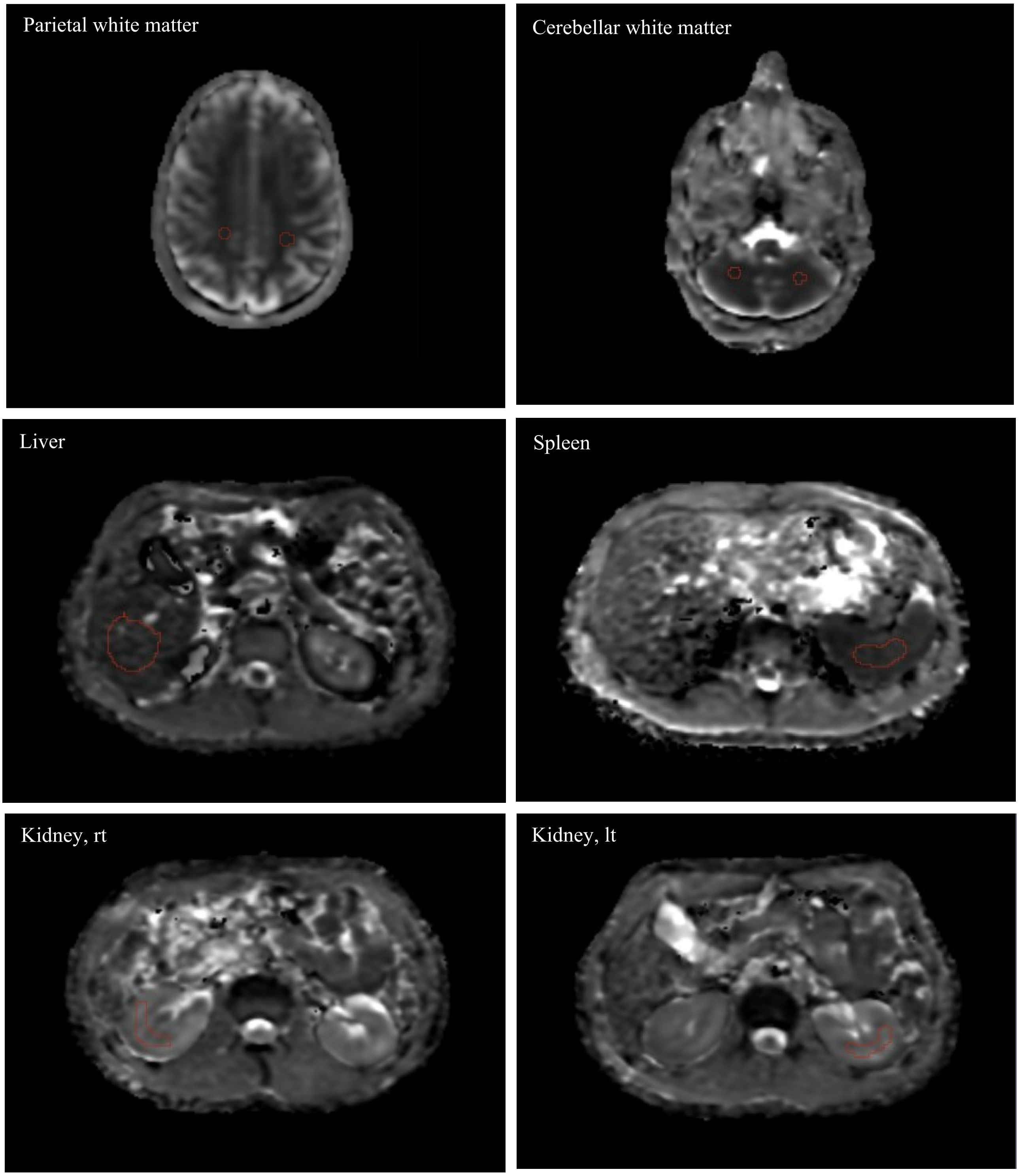

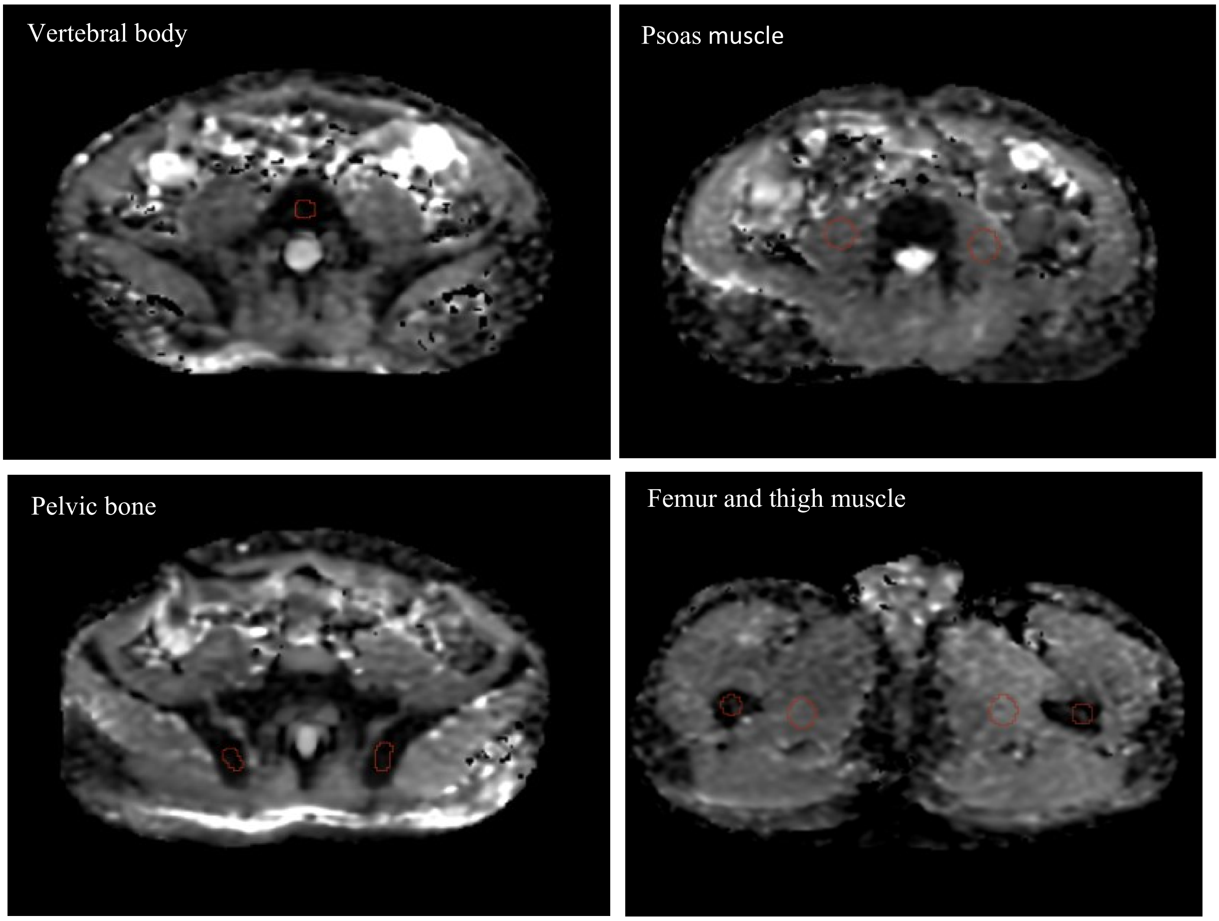
Vertebral body
Psoas muscle
Pelvic bone
Femur and thigh muscle
