# Supplementary material for: A whole-body diffusion MRI normal atlas: development, evaluation and initial use
Source: Cancer Imaging. 2023 Sep 14;23:87. doi: 10.1186/s40644-023-00603-5 (PMC10503210; doi:10.1186/s40644-023-00603-5)
Supplement: Supplementary file 6 — Supplementary Material 6. Additional file 6 is a table of ADCmedian measured in 10 tissue across the whole body (AdditionalFile6.pdf) [file 40644_2023_603_MOESM6_ESM.pdf]

Additional file 6. ADC_{median} (10^{-3} mm^2/s) measured in 10 tissues across the whole body at 1.5T (top) and 3T (bottom). The median is tabulated with the interquartile range in parenthesis.

1.5T

	ADC_{median} all (n=38)	ADC_{median} M (n=21)	ADC_{median} F (n=17)
Parietal WM	0.75 (0.057)	0.75 (0.053)	0.76 (0.060)
Cerebellar WM	0.66 (0.027)	0.67 (0.032)	0.66 (0.025)
Liver	0.59 (0.32)	0.56 (0.25)	0.68 (0.36)
Spleen	0.67 (0.24)	0.66 (0.22)	0.70 (0.30)
Kidney	1.94 (0.30)	1.90 (0.33)	1.95 (0.27)
Vertebral body	0.38 (0.21)	0.33 (0.22)	0.45 (0.14)
Psoas muscle	0.75 (0.30)	0.66 (0.26)	0.93 (0.28)
Pelvic bone	0.37 (0.16)	0.38 (0.15)	0.36 (0.18)
Femur	0.27 (0.16)	0.20 (0.12)	0.37 (0.20)
Thigh muscle	0.87 (0.47)	0.80 (0.28)	1.12 (0.40)

3T

	ADC_{median} all (n=29)	ADC_{median} M (n=16)	ADC_{median} F (n=13)
Parietal WM	0.67 (0.080)	0.67 (0.12)	0.67 (0.10)
Cerebellar WM	0.70 (0.066)	0.72 (0.085)	0.70 (0.047)
Liver	1.20 (0.29)	1.16 (0.30)	1.28 (0.34)
Spleen	0.83 (0.38)	0.83 (0.25)	0.98 (0.46)
Kidney	2.01 (0.28)	2.07 (0.28)	1.94 (0.33)
Vertebral body	0.32 (0.093)	0.33 (0.13)	0.31 (0.084)
Psoas muscle	1.42 (0.11)	1.38 (0.11)	1.45 (0.092)
Pelvic bone	0.36 (0.098)	0.33 (0.11)	0.37 (0.11)
Femur	0.37 (0.069)	0.36 (0.078)	0.39 (0.060)
Thigh muscle	1.57 (0.15)	1.54 (0.11)	1.64 (0.13)

M, male; F, female; WM, white matter
