# Supplementary material for: A whole-body diffusion MRI normal atlas: development, evaluation and initial use
Source: Cancer Imaging. 2023 Sep 14;23:87. doi: 10.1186/s40644-023-00603-5 (PMC10503210; doi:10.1186/s40644-023-00603-5)
Supplement: Supplementary file 7 — Supplementary Material 7. Additional file 7 contains a figure showing the multiple linear regression fit to tissue ADCmean, with age and sex as explanatory variables, and p > 0.05 (AdditionalFile7.pdf) [file 40644_2023_603_MOESM7_ESM.pdf]

Additional file 7. Multiple linear regression fit to tissue ADC_{mean}, with age and sex as explanatory variables. Non-significant results ($p>0.05$) are shown for 1.5T (a-d) and 3T (e-l). For each tissue type, R^2, model p-value, p-value for age, p-value for sex (female=0, male=1) and regression equation are shown. Male and female measurement points are indicated in grey and black, respectively. Regression lines are shown with sex kept constant, with the grey lines corresponding to males and the black lines to females.

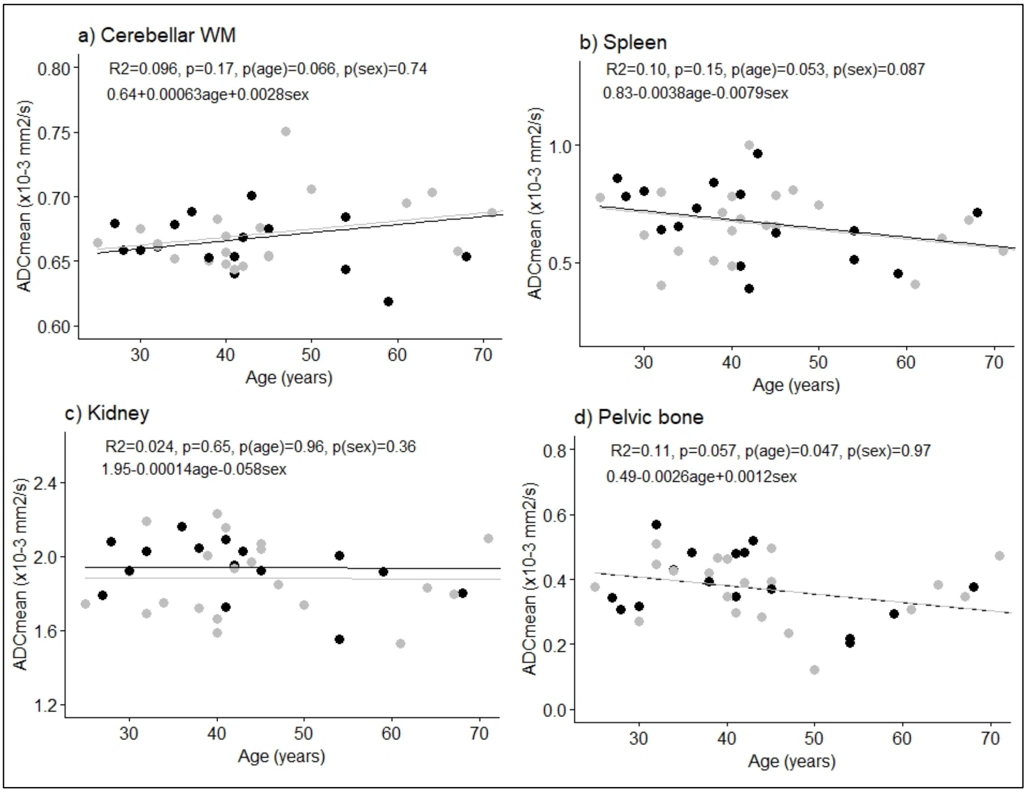

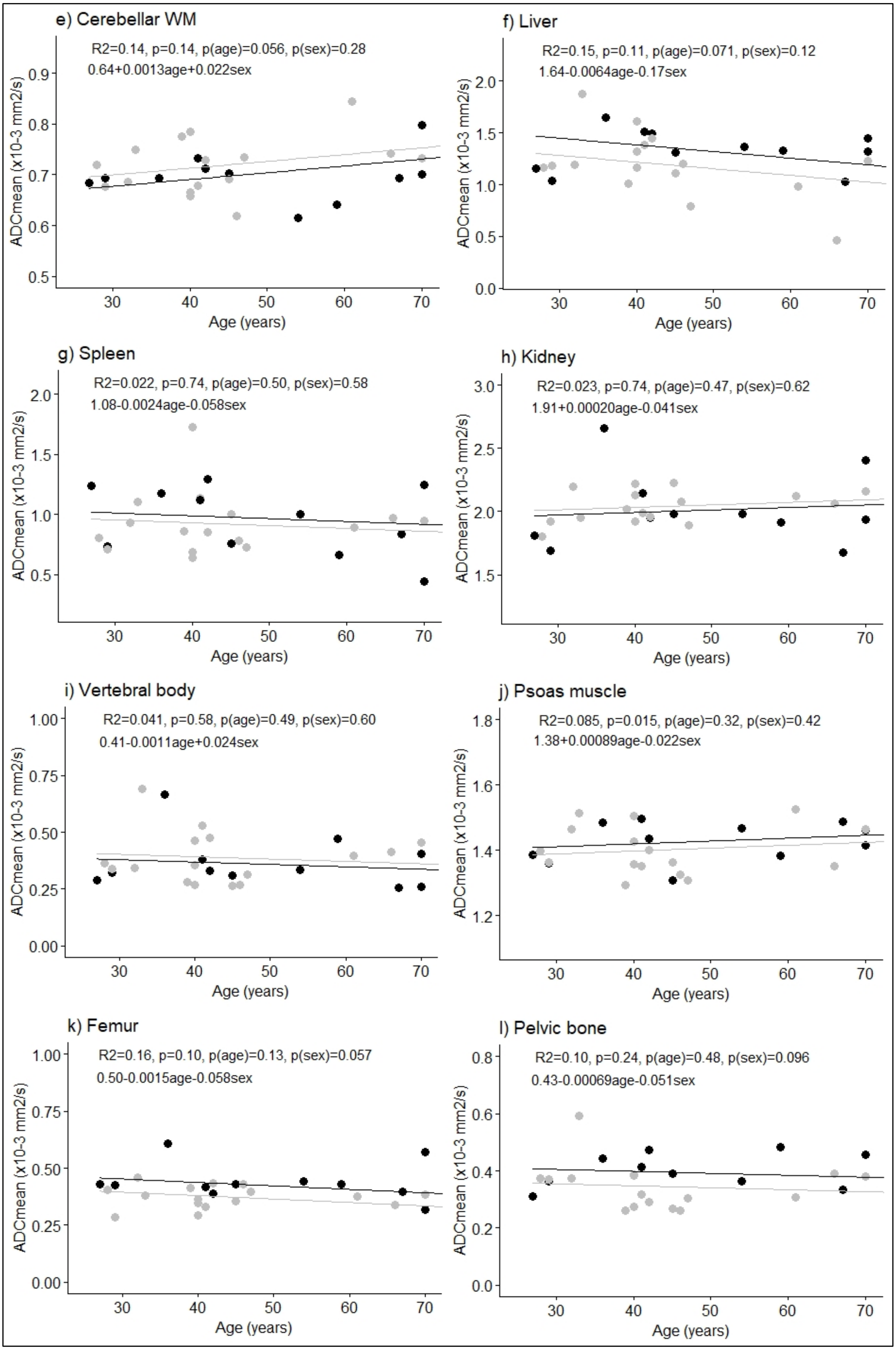
e) Cerebellar WM
R2=0.14, p=0.14, p(age)=0.056, p(sex)=0.28
0.64+0.0013age+0.022sex
f) Liver
R2=0.15, p=0.11, p(age)=0.071, p(sex)=0.12
1.64-0.0064age-0.17sex
g) Spleen
R2=0.022, p=0.74, p(age)=0.50, p(sex)=0.58
1.08-0.0024age-0.058sex
h) Kidney
R2=0.023, p=0.74, p(age)=0.47, p(sex)=0.62
1.91+0.00020age-0.041sex
i) Vertebral body
R2=0.041, p=0.58, p(age)=0.49, p(sex)=0.60
0.41-0.0011age+0.024sex
j) Psoas muscle
R2=0.085, p=0.015, p(age)=0.32, p(sex)=0.42
1.38+0.00089age-0.022sex
k) Femur
R2=0.16, p=0.10, p(age)=0.13, p(sex)=0.057
0.50-0.0015age-0.058sex
l) Pelvic bone
R2=0.10, p=0.24, p(age)=0.48, p(sex)=0.096
0.43-0.00069age-0.051sex
ADCmean (x10-3 mm2/s)
Age (years)
