# Supplementary material for: A whole-body diffusion MRI normal atlas: development, evaluation and initial use
Source: Cancer Imaging. 2023 Sep 14;23:87. doi: 10.1186/s40644-023-00603-5 (PMC10503210; doi:10.1186/s40644-023-00603-5)

Additional file 8. Whole-body coronal R-maps showing voxel-wise correlation between ADC and age for male and female subjects at 1.5T and 3T. Distortion corrected ADC data was used to produce the plots.

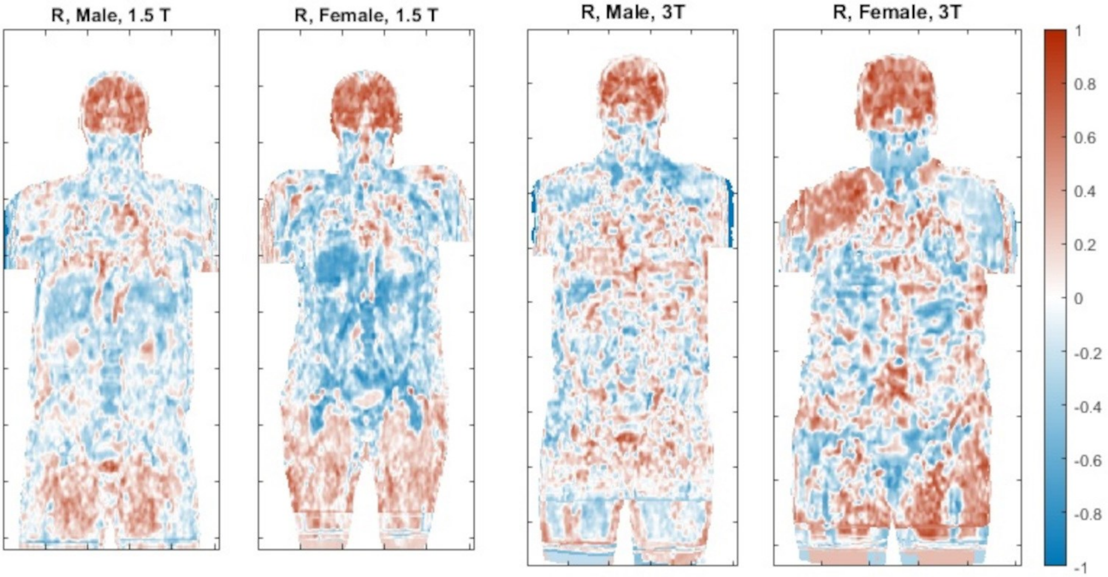

Supplement: Supplementary file 8 — Supplementary Material 8. Additional file 8 shows example whole-body R-maps for male and female subjects at 1.5T and 3T (AdditionalFile8.pdf) [file 40644_2023_603_MOESM8_ESM.pdf]
