# Supplementary material for: A whole-body diffusion MRI normal atlas: development, evaluation and initial use
Source: Cancer Imaging. 2023 Sep 14;23:87. doi: 10.1186/s40644-023-00603-5 (PMC10503210; doi:10.1186/s40644-023-00603-5)

Additional file 9. Bland-Altman plots comparing tissue ADC_{mean} measured at 1.5T and 3T for 23 dually scanned healthy volunteers.

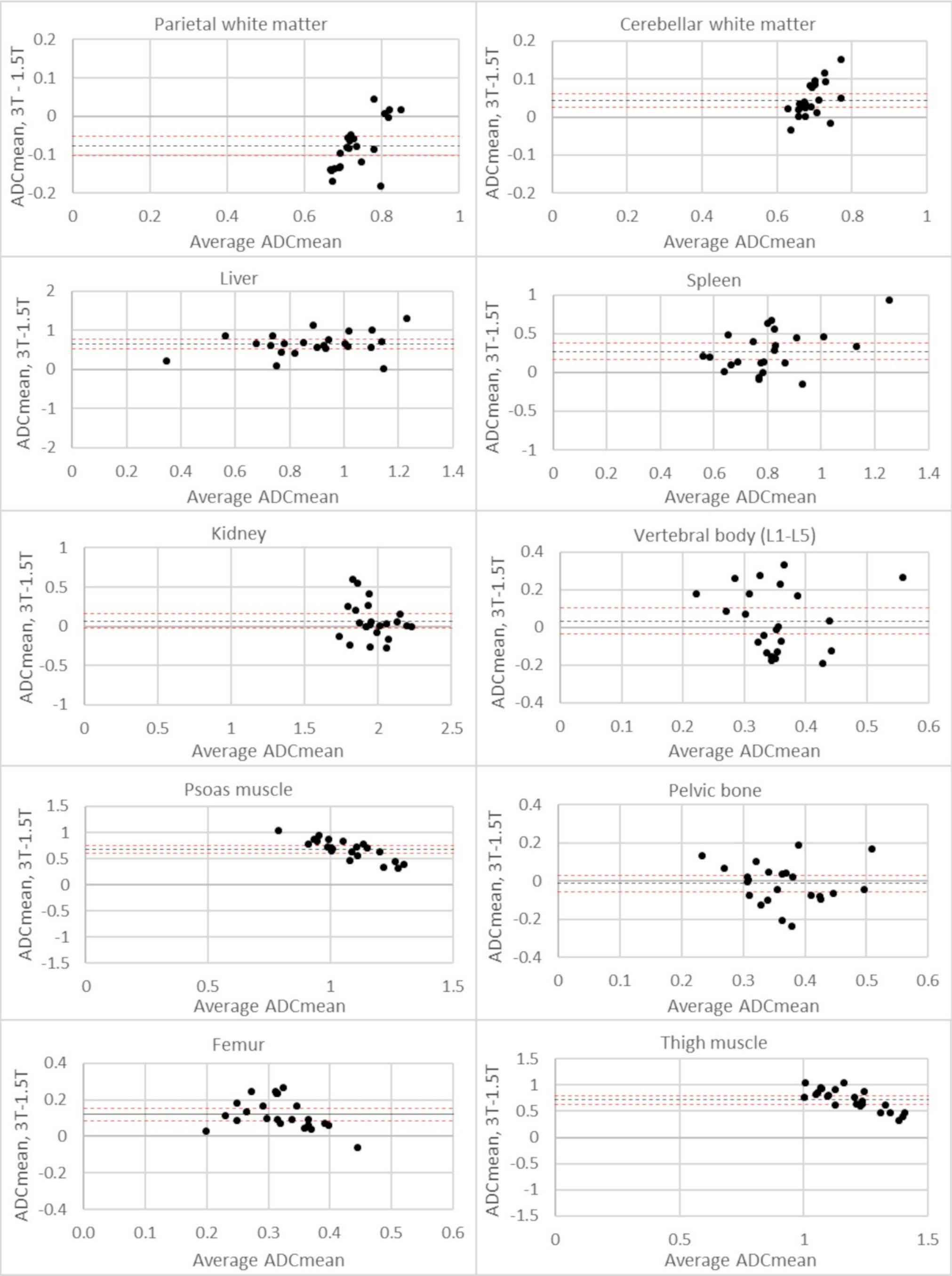

Supplement: Supplementary file 9 — Supplementary Material 9. Additional file 9 contains Bland-Altman plots for dually scanned healthy volunteers at 1.5T and 3T (AdditionalFile9.pdf) [file 40644_2023_603_MOESM9_ESM.pdf]
