# Supplementary material for: A whole-body diffusion MRI normal atlas: development, evaluation and initial use
Source: Cancer Imaging. 2023 Sep 14;23:87. doi: 10.1186/s40644-023-00603-5 (PMC10503210; doi:10.1186/s40644-023-00603-5)
Supplement: Supplementary file 10 — Supplementary Material 10. Additional file 10 shows predicted tumours overlayed on b = 900 s/mm2 MIPs (AdditionalFile_10.pdf) [file 40644_2023_603_MOESM10_ESM.pdf]

Additional file 10. Maximum intensity projection (MIP) b=900 s/mm^2 images of lymphoma patient scans (n=40) with predicted tumours highlighted for the 2 input channels (top) and 3 input channels (bottom) 3D U-Net. True positive voxels are shown in green, false negative voxels are shown in red and false positive voxels are shown in blue. MIPs are shown in inverted grey scale. The Dice score of each prediction is shown on top of each MIP image.

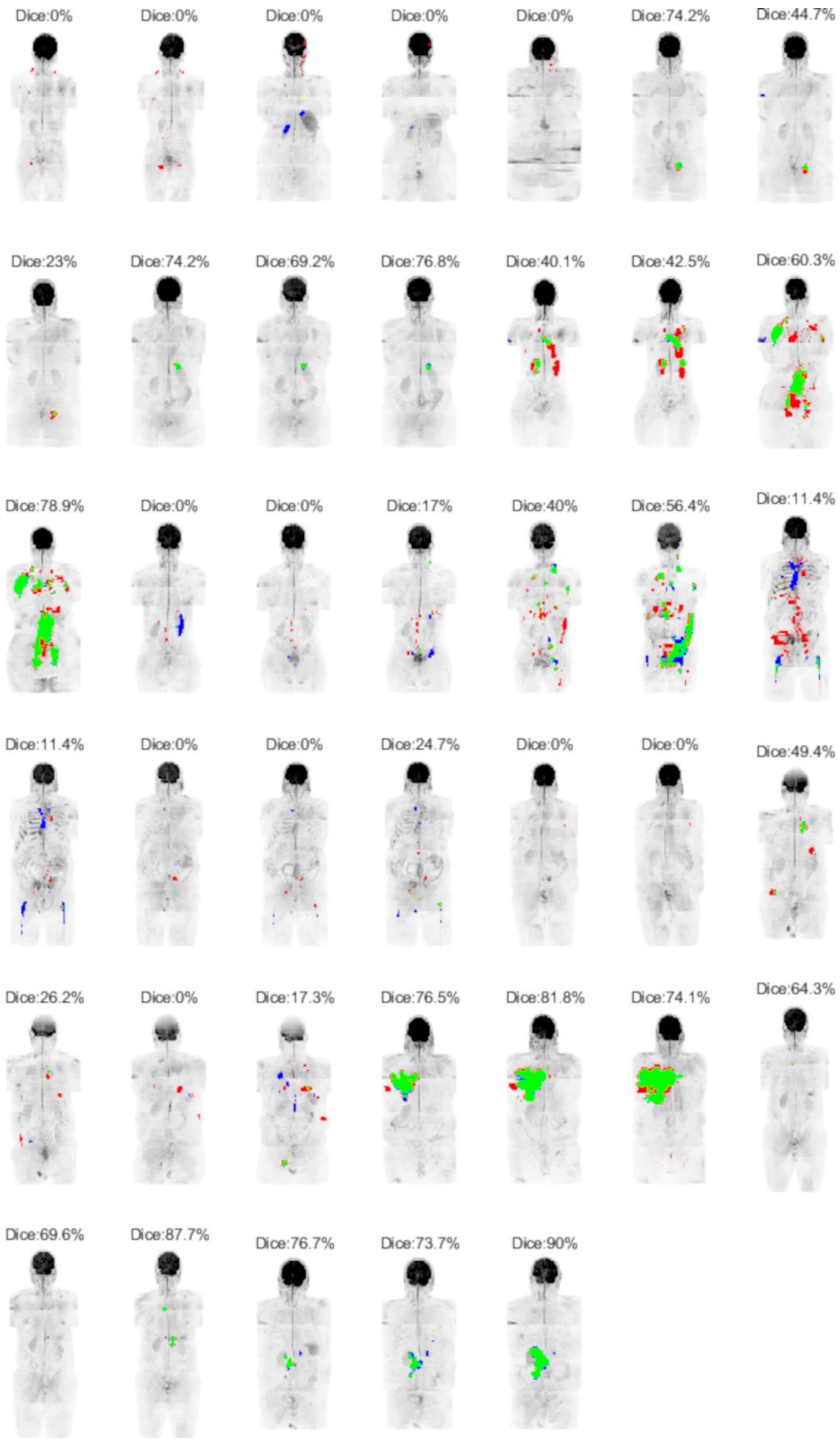
Dice:0%
Dice:0%
Dice:0%
Dice:0%
Dice:0%
Dice:74.2%
Dice:44.7%
Dice:23%
Dice:74.2%
Dice:69.2%
Dice:76.8%
Dice:40.1%
Dice:42.5%
Dice:60.3%
Dice:78.9%
Dice:0%
Dice:0%
Dice:17%
Dice:40%
Dice:56.4%
Dice:11.4%
Dice:11.4%
Dice:0%
Dice:0%
Dice:24.7%
Dice:0%
Dice:0%
Dice:49.4%
Dice:26.2%
Dice:0%
Dice:17.3%
Dice:76.5%
Dice:81.8%
Dice:74.1%
Dice:64.3%
Dice:69.6%
Dice:87.7%
Dice:76.7%
Dice:73.7%
Dice:90%

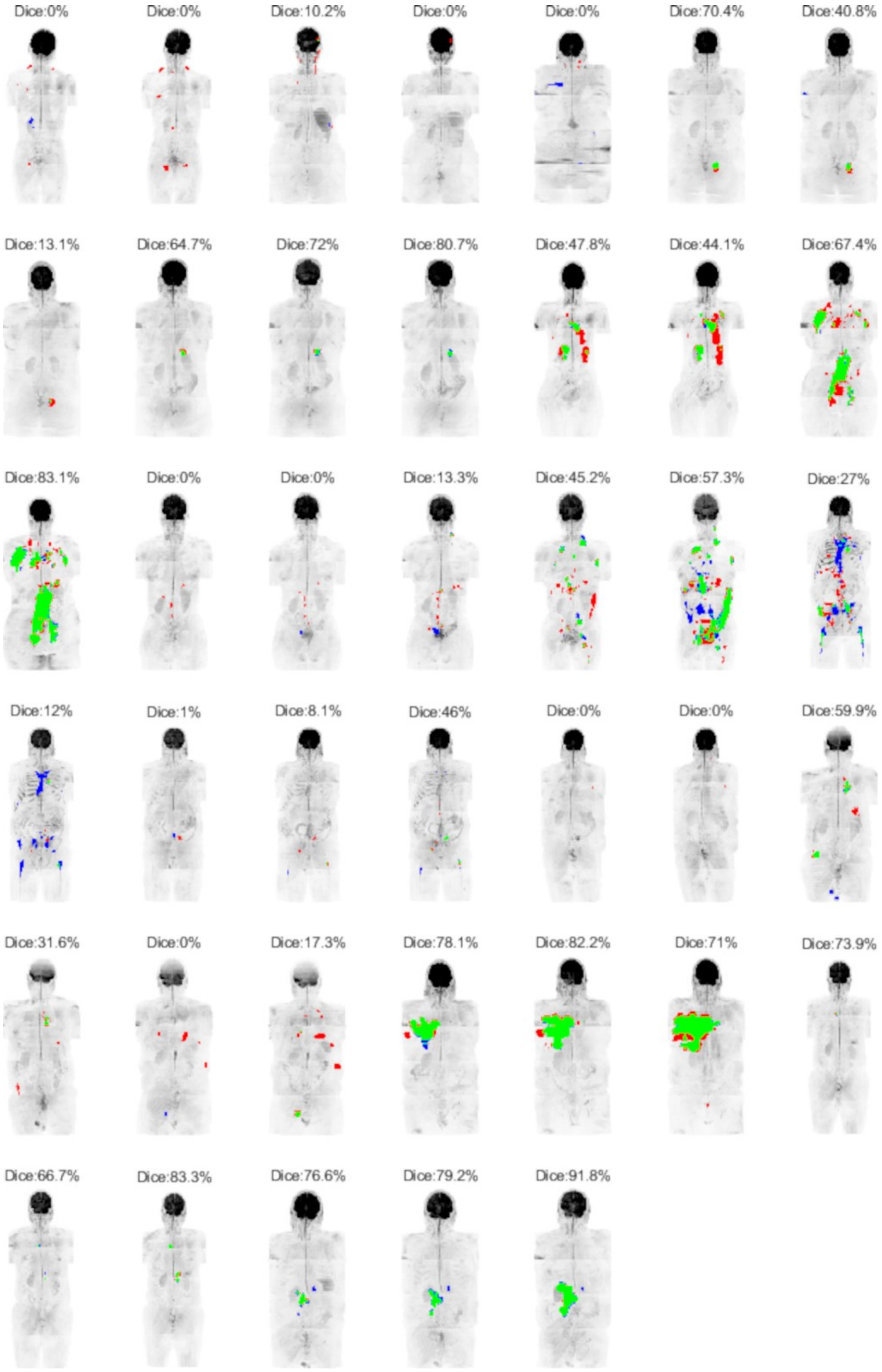
Dice:0%
Dice:0%
Dice:10.2%
Dice:0%
Dice:0%
Dice:70.4%
Dice:40.8%
Dice:13.1%
Dice:64.7%
Dice:72%
Dice:80.7%
Dice:47.8%
Dice:44.1%
Dice:67.4%
Dice:83.1%
Dice:0%
Dice:0%
Dice:13.3%
Dice:45.2%
Dice:57.3%
Dice:27%
Dice:12%
Dice:1%
Dice:8.1%
Dice:46%
Dice:0%
Dice:0%
Dice:59.9%
Dice:31.6%
Dice:0%
Dice:17.3%
Dice:78.1%
Dice:82.2%
Dice:71%
Dice:73.9%
Dice:66.7%
Dice:83.3%
Dice:76.6%
Dice:79.2%
Dice:91.8%
